# T-cell tolerance and exhaustion in the clearance of *Echinococcus multilocularis*: role of inoculum size in a quantitative hepatic experimental model

**DOI:** 10.1038/s41598-017-11703-1

**Published:** 2017-09-11

**Authors:** Chuanshan Zhang, Yingmei Shao, Shuting Yang, Xiaojuan Bi, Liang Li, Hui Wang, Ning Yang, Zhide Li, Cheng Sun, Liang Li, Guodong Lü, Tuerganaili Aji, Dominique A. Vuitton, Renyong Lin, Hao Wen

**Affiliations:** 1 State Key Laboratory Incubation Base of Xinjiang Major Diseases Research, and WHO Collaborating Centre on Prevention and Case Management of Echinococcosis, Clinical Medicine Institute, The First Affiliated Hospital of Xinjiang Medical University, Urumqi, Xinjiang China; 2 Department of Hepatic Hydatid and Hepatobiliary Surgery, Digestive and Vascular Surgery Centre, The First Affiliated Hospital of Xinjiang Medical University, Urumqi, Xinjiang China; 3 Xinjiang Key Laboratory of Echinococcosis, Clinical Medicine Institute, The First Affiliated Hospital of Xinjiang Medical University, Urumqi, Xinjiang China; 4 Institute of Immunology, The Key Laboratory of Innate Immunity and Chronic Disease (Chinese Academy of Medical Science), School of Life Sciences and Medical Center, University of Science & Technology of China, Hefei, Anhui China; 5 WHO-Collaborating Centre for the Prevention and Treatment of Human Echinococcosis, Department of Parasitology, University Bourgogne Franche-Comté (EA 3181) and University Hospital, Besançon, France

## Abstract

The local immune mechanisms responsible for either self-healing or sustained chronic infection are not clear, in the development of *E. multilocularis* larvae. Here, we developed a suitable experimental model that mimics naturally infected livers, according to the parasite load. We demonstrated that local cellular immunity and fibrogenesis are actually protective and fully able to limit metacestode growth in the liver of low or medium dose-infected mice (LDG or MDG), or even to clear it, while impairment of cellular immunity is followed by a more rapid and severe course of the disease in high dose-infected mice (HDG). And recruitment and/ or proliferation of memory T cells (including CD4 Tem, CD8 Tcm and CD8 Tem) and imbalance of T1/T2/T17/Treg-type T cells in liver were not only associated with clearance of the parasite infection in LDG, but also with increased hepatic injury in HDG; in particular the dual role of CD8 T cells depending on the parasite load and the various stages of metacestode growth. Besides, we first demonstrate the association between LAG3- or 2B4-expressing T cells exhaustion and HD inocula in late stages. Our quantitative experimental model appears fully appropriate to study immunomodulation as a therapeutic strategy for patients with Alveolar Echinococcosis.

## Introduction

The larval stage of the fox-tapeworm *Echinococcus (E.) multilocularis* is the causative agent of hepatic alveolar echinococcosis (AE), one of the most dangerous parasitic diseases of the northern hemisphere^1^. AE is characterized by an infiltrative, destructive and tumor-like growth of the *E. multilocularis* metacestode, and a granulomatous host reaction resulting from the liver homing of cells involved in the immune response^2^. That immune response which develops against the larval stages of *E. multilocularis* accounts for a controlled parasite tissue development, but also for immunopathological events, eventually leading to liver fibrosis and necrosis^3^.

In AE patients, depending on the type of immune response elicited by the host, infection will have different clinical presentations: (1) “resistant AE patients”, without chronic infection, and either no lesions, or only “dying” or “aborted” lesions; (2) “susceptible AE patients”, with slow growth of the metacestode and chronic infection, and (3) “highly susceptible AE patients”, with uncontrolled and rapid metacestode proliferation, as it occurs in individuals with impaired immunity. It is suggested that in those individuals where infection leads to disease, the developing parasite is partially controlled by host’s immunity^4–6^. Moreover, impairment of local and systemic immune regulation may explain the persistence of cellular infiltration and fibrogenesis in patients with clinically expressed AE. However, the mechanisms responsible for either self-healing or maintenance of a chronic infection are not very clear.

The conceptual consequences of these findings in AE patients, cover two complementary, assessments: (1) natural (immunological) mechanisms of defense (innate and/or acquired) are at work in the majority of human hosts, which are able to stop the larval growth at the very first stages or after the beginning of its development in the liver; (2) strategies are operating at the parasite’s level, which may counteract the immune system of the host and even take advantage of it for its own growth and survival in the liver^3^. In murine alveolar echinococcosis and in AE patients as well, little is known about the relationship between the dose of injected metacestode, host immune response and self-healing/maintenance of a chronic infection. In AE patients, the initial parasite load is always unknown; so this relationship cannot be studied. Host-parasite interactions may be studied by using a model of primary infection of intermediate hosts, after ingestion of *E. multilocularis* eggs^7^; however, in addition to being at risk for the operator, the route of infection involves numerous host-dependent steps and the outcome is also dependent on non-immunological events, such as gastric and enteric enzymes, bile composition, or nature of the intestinal barrier. It is the reason why host-parasite immunological relationship has usually been investigated experimentally using secondary AE, in which homogenates of the larval parasite are injected in the peritoneum^8^, in the subcutaneous space^9^ or directly in the liver^10^ of animal intermediate hosts. These routes of infection are widely used because they are relatively easy and safe, but the first two models do not reproduce the ‘natural’ location of the initial development of the parasite (i.e. the liver), and with the third model an accurate control of the extent of liver infection is difficult. As protoscoleces (PSCs), which in the parasite cycle transform into adult worms in the definitive hosts, are also able to differentiate into metacestode, direct injection of precise numbers of PSCs in the portal vein could overcome the usually encountered difficulties and make us able to characterize the systemic but also local, hepatic, immune mechanisms which either clear *E. multilocularis* larvae from the liver or maintain a chronic infection, and to assess the influence of parasite load on these mechanisms.

From various studies performed in AE patients and in experimental models, it is commonly accepted that metacestode persistence is the consequence of immune tolerance, mainly mediated by specialized regulatory T cells and related cytokines such as IL-10 and TGF-β^11, 12^. Persistent infection leads also to a disruption of the normal immunodominance hierarchy and function of T cell responses which is referred to as ‘functional exhaustion’. T cell exhaustion occurs by a stepwise loss of function due to high antigen load. Cells first lose cytotoxic abilities, then production of effector cytokines, such as IFN-γ, TNF-α and granzyme B. Antigens from the infectious agent appear to be the driving force for this loss of function, since a strong correlation exists between the dose of inoculum and the level of exhaustion. Effector T cell exhaustion was described in humans in the context of chronic viral infections, parasitic infections as well as liver cancer^13^. This type of mechanism has, however, received no attention in *E. multilocularis* infection, despite its chronic nature and high antigen load. Up-regulation of multiple inhibitory molecules has been shown to play a major role in this process^14^, including molecules encoded by inhibitory lymphocyte activation gene-3 (LAG3) and 2B4. Sustained signaling through these inhibitory receptors directly and indirectly induce transcriptional changes that negatively regulate cell proliferation and expression of proinflammatory cytokines by T cells^15, 16^.

In this study, we first developed an experimental mouse model by injection of *E. multilocularis* PSCs via the portal vein. Then, we investigated the impact of the inoculation of different PSC numbers (corresponding to low, medium and high dose groups, respectively LDG, MDG and HDG) on the development of the metacestode in the liver of C57/B6 mice after infection, and we assessed the relationship between the observed histological type and intensity of infection and the liver damage, as assessed by fibrosis deposits in the liver. To study the relationship between infection following increasing number of PSCs and the host’s immune response, and make the immunological profile observed in this model comparable to that described in other experimental models of *E. multilocularis* infection, we measured most of the known cellular and cytokine markers of the immune balance. In addition, we tested the hypothesis that the exhaustion phenomenon plays a role in the differential growth of the metacestode after infection by different amounts of inoculum by measuring LAG3 and 2B4 exhaustion markers and testing the functional capacity of the T cells which expressed these inhibitory molecules.

## Results

### Establishment of a mouse model by injecting increasing number of *E. multilocularis* PSCs via the portal vein

All animals tolerated the procedure well and survived in the whole period of infection. In a first set of experimental infections, we compared the susceptibility of C57/B6 mice known to be moderately susceptible/resistant to *E. multilocularis* infection to the injection of low dose (LD, 50, 250), medium dose (MD, 500), and high dose (HD, 1000, 2000) of live PSCs via the portal vein.

The analysis of the lesions at the surface of the liver, and the number of metastases ranging from 2 to 24 weeks after infection, showed a different course of infection in the different dose groups (G) (Supplementary Fig. S1). At 2 weeks, minute yellowish grey in color and opaque liver foci less than 1 mm in size were observed macroscopically in all dose groups. At 4 weeks, small multilocular foci of 1–3 mm size were seen on the surface of the anterior lobe of the liver in HDG, while minute liver foci were observed in LDG and MDG. At 8 and 12 weeks, lesions from 1 to 6 mm in diameter and 1–8 in number were found in the lateral and caudate lobe of the liver in HDG, and in only 1/5 mice in MDG, while no or very tiny lesions were observed in LDG. At 16 weeks, lesions from 3 to 7 mm in diameter and 1–6 in number were found in the liver in all mice of HDG, and in only 1/5 mice in MDG, while no or tiny lesions were still observed in LDG. At 20 and 24 weeks, numerous lesions from 4 to 20 mm in diameter were found in the liver, and almost the entire liver was replaced by metacestode tissue in all mice of HDG. Only 2/9 mice of MDG had such lesions; but no liver lesions were seen in LDG.

### Histopathology and types of hepatic lesions in mice with different PSC inocula

**Figure 1:**
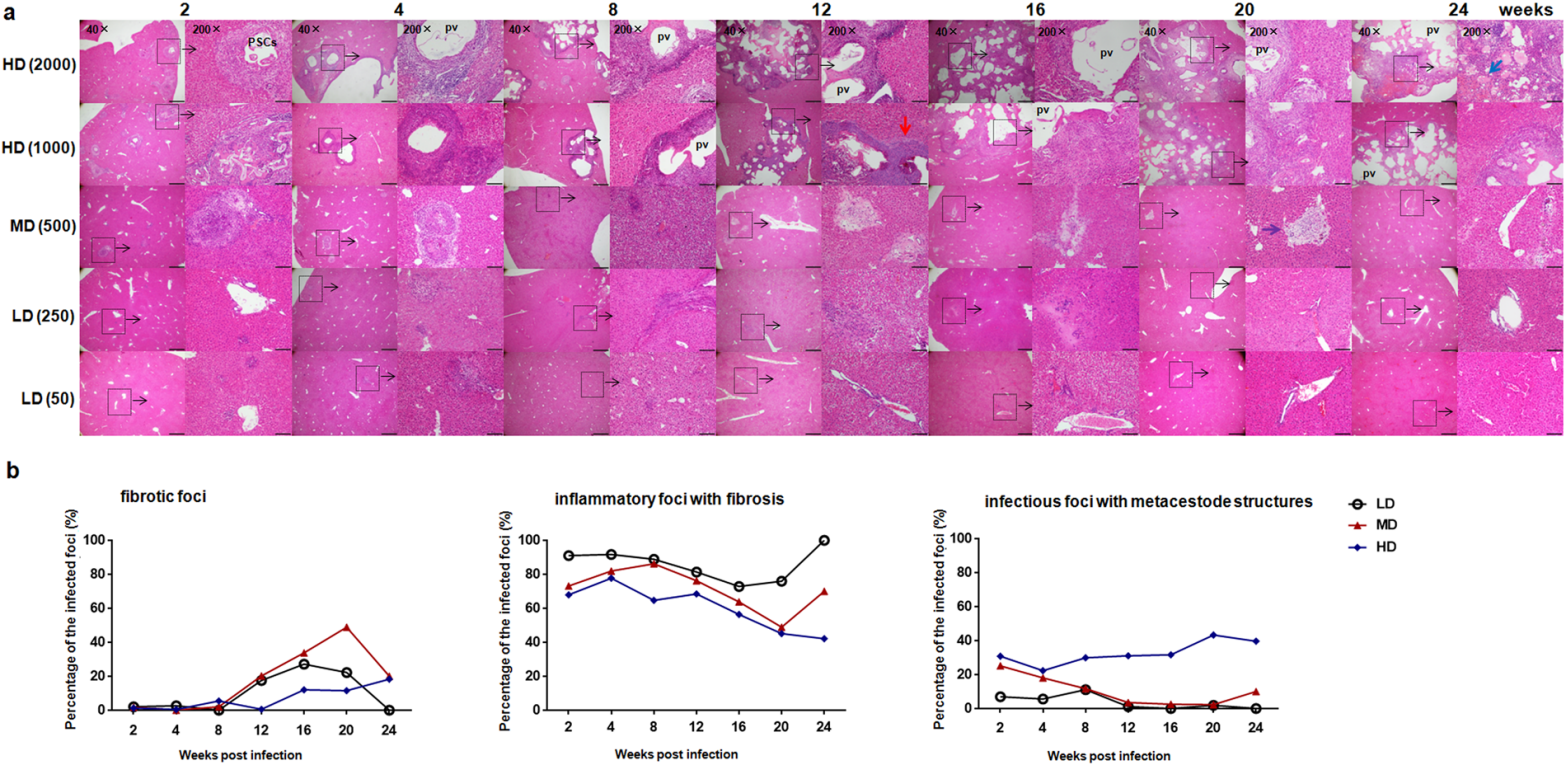
Hepatic histopathological alterations and granulomatous response in mice infected with different *E. multilocularis* PSC inocula during course of infection. (**a**) Histopathological alterations of the liver from mice infected with different PSC inocula during course of infection. H&E staining of liver sections. The original magnification was at 40×, and the right corresponding images were magnified at 200×; bars indicate 500 μm or 100 μm in the 40× or in the 200× magnification images, respectively. Red arrow indicates inflammatory cell zone; Blue arrow indicates coagulation necrosis areas; Purple arrow indicates fibrosis areas. Dashed line marks border of granuloma around parasitic lesion. (**b**) Hepatic granulomatous response to *E. multilocularis* infection with different doses. Liver histological reaction at each infectious foci was scored as (1) ‘fibrotic foci’: parasite-free, no visible PSCs or metacestode, only liver fibrosis; (2) ‘inflammatory foci with fibrosis’: parasite-free, no visible PSCs or metacestode, small inflammatory foci with liver fibrosis; (3) ‘infectious foci with metacestode structures’: fibroblasts, myofibroblast, lymphocytes and monocytes surrounding parasitic lesion with identified germinal layer. Almost 100 infectious foci were identified microscopically in liver sections from 5–6 mice per group. PSCs: protoscoleces. LD: 50 or 250 PSCs; MD: 500 PSCs; HD: 1000 or 2000 PSCs. pv: parasitic vesicle. Data are shown as mean ± standard error of the mean (SEM).

In experimental mice, at 2 weeks, only infiltrating lymphocytes surrounding the *E. multilocularis* inoculum could be observed in all infected groups, and a few PSCs from the hepatic portal vein inoculum were still present in HDG. From 4 to 12 weeks, at the periphery of the lesion(s), numerous fibroblasts and inflammatory cells were present and an obvious increase of liver fibrosis was observed. In addition, characteristic *E. multilocularis* lesions appeared in HDG. However, the germinal layer of *E. multilocularis* metacestode could not be identified in LDG and MDG. From 16 to 24 weeks, in HDG, the pseudo-tumor parasitic mass included many vesicular structures embedded within thick fibrous tissue, and the periparasitic area was composed of inflammatory fibrous tissue and necrotic areas intermixed with immune cell infiltrate including small granulomatous nodules. The hepatic parenchyma close to the lesions was progressively invaded by fibrous connective tissue septa, and solitary islands of hepatic tissue were observed. In MDG, only 3/14 mice livers exhibited parasitic lesions, while the other mice livers exhibited only inflammatory infiltrates and liver fibrosis. In LDG, lymphocyte clusters with mild fibrogenesis were also, but more rarely, disclosed within the hepatic lobules and in portal areas; all parasitic foci were cleared (Fig. 1a). Mice in the control group at the same time-points showed normal hepatic histology (data not shown; available from reference^17^).

The histological response to *E. multilocularis* infection (i.e. ‘infectious foci’) was classified into three categories: infectious foci with metacestode structures, inflammatory foci with fibrosis, and fibrotic foci. As shown in Fig. 1b, in LDG, inflammatory foci with fibrosis were the most frequently observed lesions at each time points (100% of all ‘infectious foci’ at 24 weeks), while infectious foci with metacestode structures were almost never found in the liver. After 8 weeks of infection, fibrotic foci gradually increased in LD mice and then peaked at 16 weeks. In MDG, the number of inflammatory foci with fibrosis gradually increased and peaked at 8 weeks (86.2% of all infectious foci), and then decreased from 12 to 20 weeks. Fibrotic foci gradually increased after 8 weeks of infection, and peaked at 20 weeks, representing 48.8% of all infectious foci; they then decreased at 24 weeks (20.0% of all infectious foci). There were very few infectious foci with metacestode structures after 8 weeks infection. In HDG, the infectious foci with metacestode structures gradually increased after 4 weeks, and peaked at 20 weeks (43.3% of all infected foci). The inflammatory foci with fibrosis gradually decreased after 4 weeks, and reached their lowest level at 24 weeks (42.1% of all infectious foci). In addition, the percentage of fibrotic foci gradually increased from 12 to 24 weeks, but still remained a very small proportion of all infected foci at 24 weeks (18.3% of all infectious foci).

Based on macroscopic and microscopic observations described above, and to further explore the correlation between inoculum doses, development of parasite-induced immune response, and resistance to infection, we selected the groups inoculated with 50 (LDG), 500 (MDG) and 2000 (HDG) PSCs to study their immune response at 2 (early stage), 12 (middle stage) and 24 weeks (later stage) after infection.

### Liver lesion size and inflammatory cell infiltration in mice with different PSC inocula

**Figure 2:**
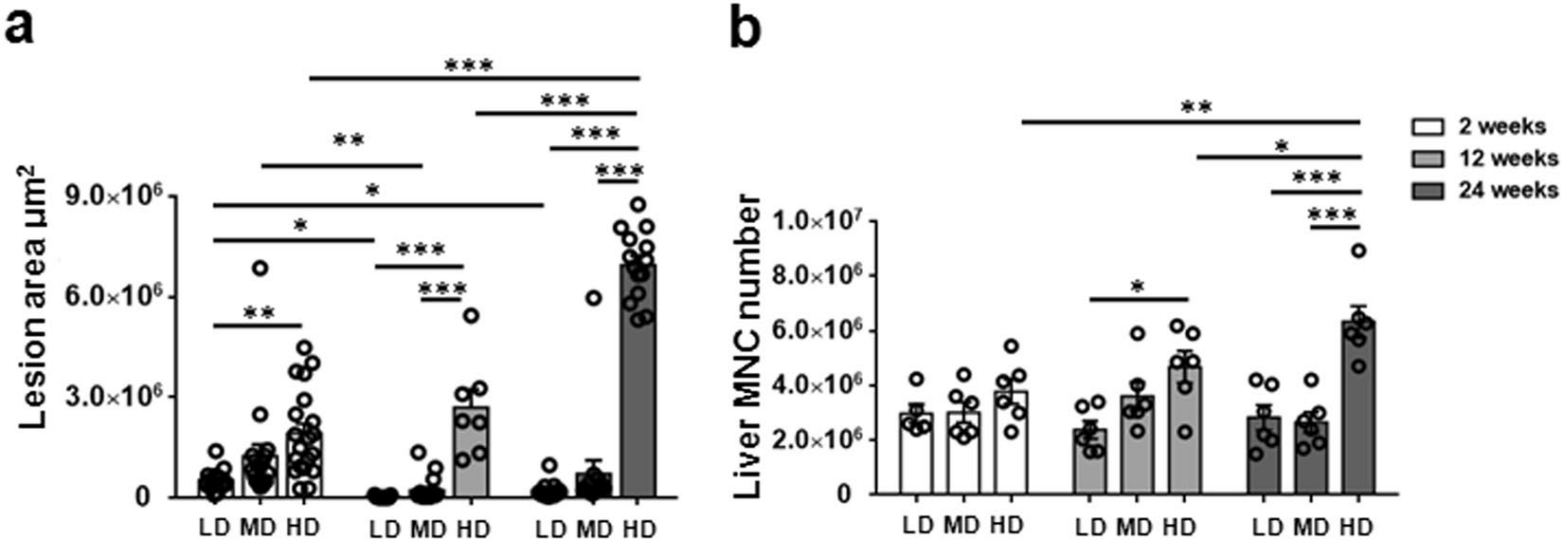
Hepatic lesion size and inflammatory infiltration in mice infected with different *E. multilocularis* PSC inocula during the course of infection. (**a**) Liver lesion area determined by microscopic measuring of H&E-stained tissue sections, and expressed as square micrometers (μm^2^). (**b**) Total number of hepatic mononuclear cells (MNCs). LD: 50 PSCs; MD: 500 PSCs; HD: 2000 PSCs. Data are shown as mean ± standard error of the mean (SEM, 5–6 mice per group), *p < 0.05, **p < 0.01 and ***p < 0.001.

In LDG and MDG, the largest average size of liver lesions was observed at 2 weeks. After 12 weeks, development of liver lesions was obviously reduced, which was consistent with parasite clearance in LGD or MGD. In HDG, the average size of liver lesions showed a faster increase and it was significantly larger than that of LDG and MDG at 2, 12 and 24 weeks (Fig. 2a). In addition, the number of mononuclear cells (MNCs) infiltrating the liver was significantly higher in HDG than in LDG or MDG at 12 and 24 weeks. However, it was no significant difference among all groups at 2 weeks (Fig. 2b).

### Characteristics of hepatic fibrosis in mice with different PSC inocula

In LDG and MDG, mild collagen deposition assessed by Sirius red staining was seen around small inflammatory foci and in the portal spaces. In HDG, the degree of staining was higher than that observed in LDG and MDG at each time points. In line with this observation, collagen 1A1 (COL1A1) mRNA expression in the liver was higher in HDG than in LDG and MDG at 2, 12 and 24 weeks.

We analyzed α-SMA expression both by immunohistochemistry (IHC) and quantitative real time-PCR (qRT-PCR). IHC showed α-SMA positive cells mainly around the infectious foci and the portal areas. At 12 weeks, the percentage of α-SMA positive cells was higher in HDG than in LDG. At 24 weeks, the percentage of α-SMA positive cells was higher in HDG than in LDG and MDG. These observations were consistent with α-SMA mRNA expression, as determined by qRT-PCR. (Supplementary Fig. S2).

### Lymphocyte composition in the liver of mice with different PSC inocula

**Figure 3:**
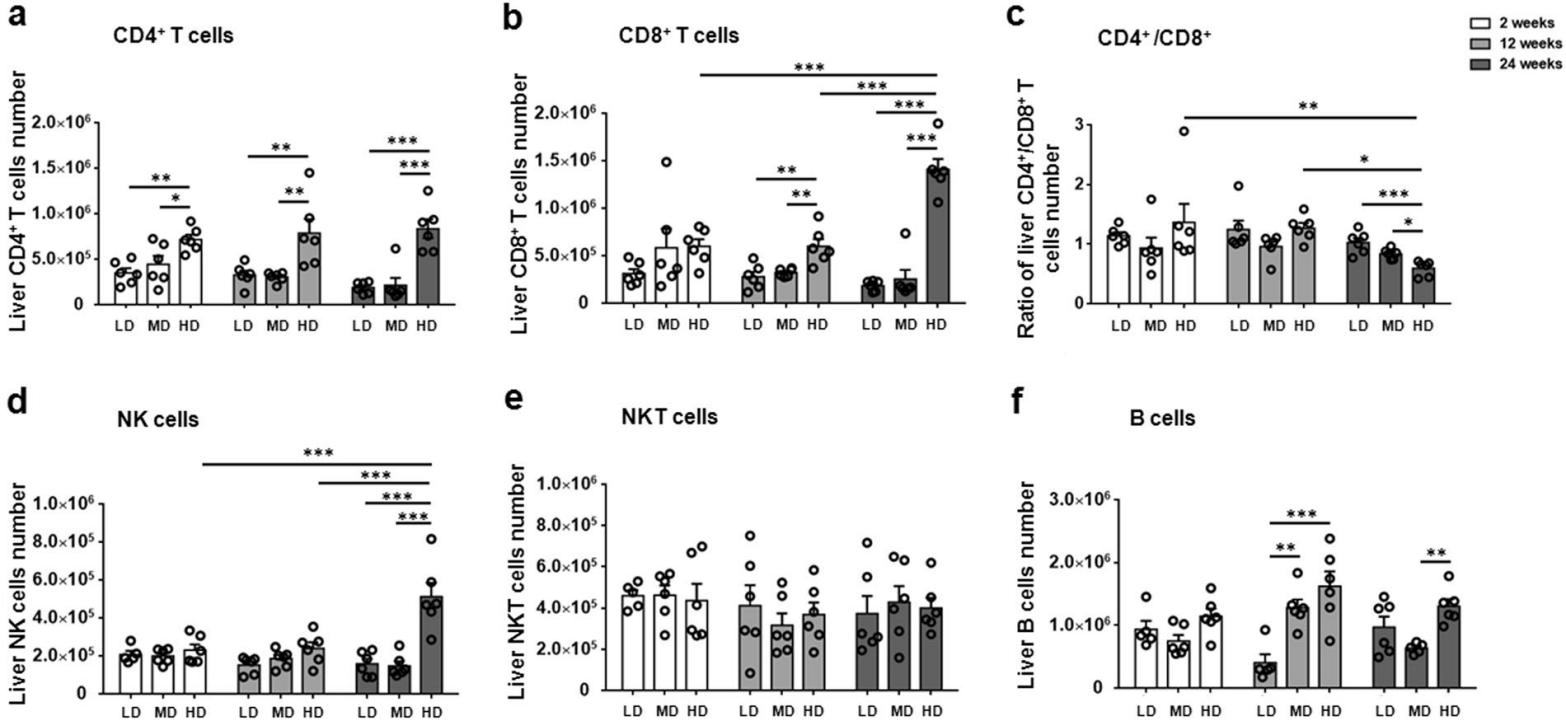
Hepatic inflammatory cell subsets in mice infected with different *E. multilocularis* PSC inocula during the course of infection. (**a**) Absolute quantification of hepatic CD4 (NK1.1^−^CD3^+^CD4^+^) T cells. (**b**) Absolute quantification of hepatic CD8 (NK1.1^−^CD3^+^CD8^+^) T cells. (**c**) The ratio of CD4 T cells / CD8 T cells in the liver. (**d**) Absolute quantification of hepatic NK (NK1.1^+^CD3^−^) cells. (**e**) Absolute quantification of hepatic NKT cells. (**f**) Absolute quantification of hepatic CD19 B cells. LD: 50 PSCs; MD: 500 PSCs; HD: 2000 PSCs. Data are shown as mean ± standard error of the mean (SEM, 5–6 mice per group), *p < 0.05, **p < 0.01 and ***p < 0.001.

The absolute numbers of CD4 T cells and of CD8 T cells were higher in HDG than in LDG and MDG at 2, 12 and 24 weeks. More importantly, the absolute number of CD8 T cells was significantly increased from 12 to 24 weeks in HDG, while the number of CD4 T cells was not significantly changed (Fig. 3a, b). The hepatic CD4/CD8 ratio gradually decreased as the infection dose increased; it was lower in HDG than in LDG and MDG at 24 weeks (p = 0.0013; p = 0.0181). Moreover, the ratio of CD4/CD8 significantly decreased from 12 to 24 weeks in HDG (p = 0.0384). (Fig. 3c).

At 24 weeks, the absolute number of NK cells was higher in HDG than in LDG and MDG, and it was significantly increased in HDG from 12 to 24 weeks (Fig. 3d). The number of NKT cells was not significantly different among all groups (Fig. 3e). In addition, the absolute number of CD19 B cells was higher in MDG and HDG than in LDG at 12 weeks; at 24 weeks it was higher in HDG than in MDG (Fig. 3f).

### Phenotype analysis of memory T cell subsets in the liver of mice with different PSC inocula

**Figure 4:**
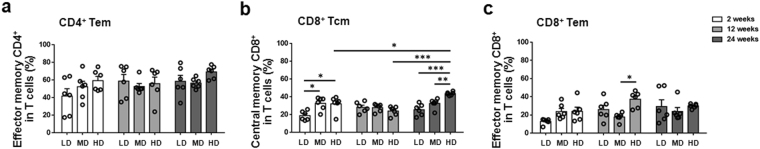
Hepatic memory T cell phenotypes in mice infected with different *E. multilocularis* PSC inocula during the course of infection. (**a**) The percentages of effector memory CD4 T cells (Tem, CD44^+^CD62L^−^) on CD4 T cells in the liver. (**b**) The percentages of central memory CD8 T cells (Tcm, CD44^+^CD62L^+^) on CD8 T cells in the liver. (**c**) The percentages of effector memory CD8 T cells (Tem, CD44^+^CD62L^−^) on CD8 T cells in the liver. LD: 50 PSCs; MD: 500 PSCs; HD: 2000 PSCs. Data are shown as mean ± standard error of the mean (SEM, 5–6 mice per group), *p < 0.05, **p < 0.01 and ***p < 0.001.

At 2 and 12 weeks, the percentages of naive CD4 T cells and of memory CD4 T cells were not significantly different. At 24 weeks, the percentage of effector memory CD4 T cells (Tem, CD44^+^CD62L^−^) was higher in HDG than in control group, and approximated 70% of CD4^+^ T cells (p = 0.0219, control data not shown). (Fig. 4a and Supplementary Fig. S3).

At 2 weeks, the percentage of central memory CD8 T cells (Tcm, CD44^+^CD62L^+^) was higher in MDG and HDG than in LDG (p = 0.0076; p = 0.0099). The percentage of effector memory CD8 T cells (Tem, CD44^+^CD62L^−^), albeit higher in MDG and HDG than in LDG, was not significantly different in the 3 groups. At 12 weeks, CD8 Tem percentage was higher in HDG than in MDG. At 24 weeks, CD8 Tcm percentage was higher in HDG than in LDG and MDG (p = 0.0001; p = 0.0033). CD8 Tem percentage was higher in HDG than in control group, but there was no significant difference among all groups (p = 0.1808). (Fig. 4b, c and Supplementary Fig. S3).

### Dynamic changes of hepatic T1-type CD4 T cells and CD8 T cells and related cytokines in mice with different PSC inoculum

**Figure 5:**
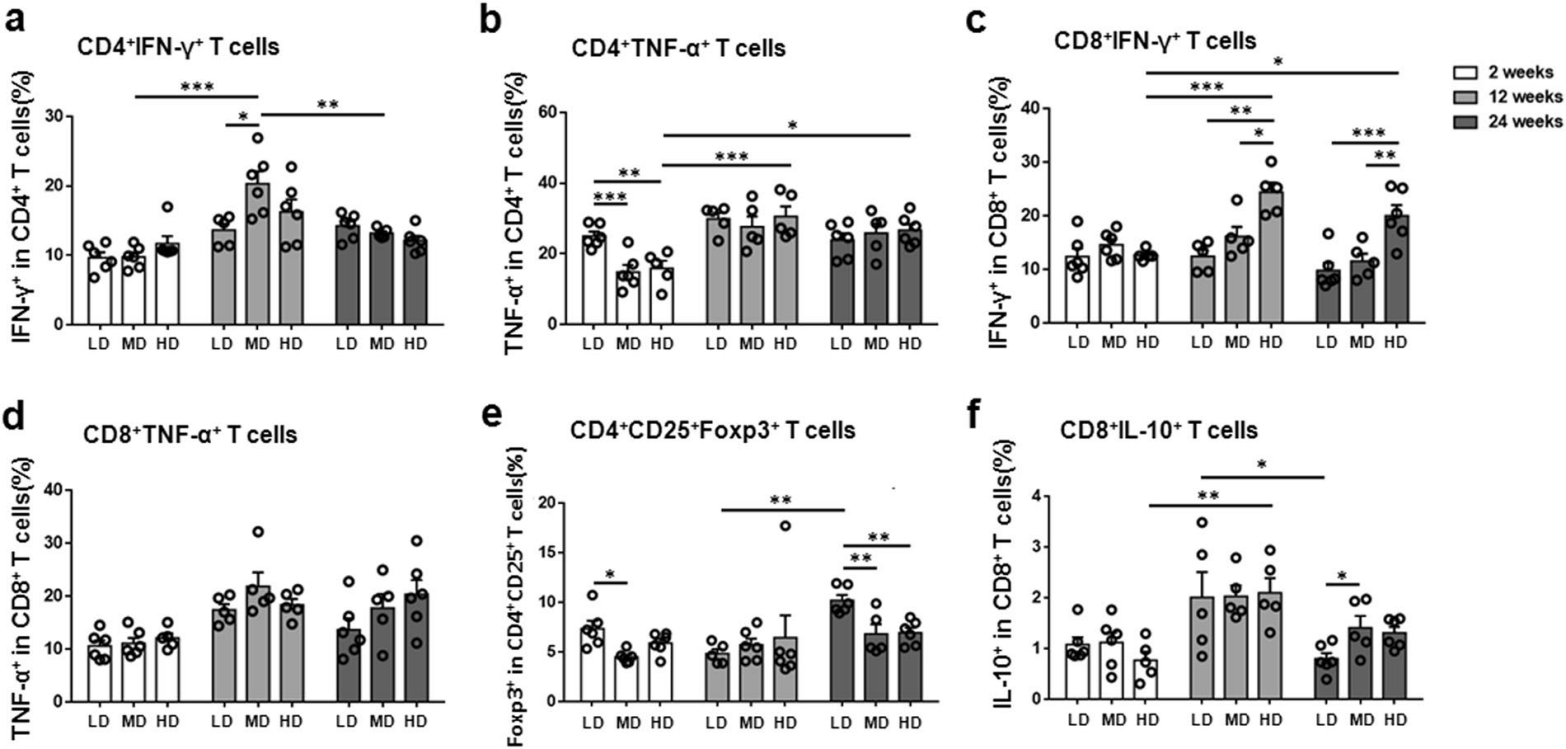
Distribution of hepatic T cell subsets in mice infected with different *E. multilocularis* PSC inocula during the course of infection. (**a**) The percentage of CD4^+^IFN-γ^+^ T cells (T1-type) on CD4 T cells in the liver. (**b**) The percentage of CD4^+^TNF-α^+^ T cells (T1-type) on CD4 T cells in the liver. (**c**) The percentage of CD8^+^IFN-γ^+^ T cells (T1-type) on CD8 T cells in the liver. (**d**) The percentage of CD8^+^TNF-α^+^ T cells (T1-type) on CD8 T cells in the liver. (**e**) The percentage of CD4^+^CD25^+^Foxp3^+^ T cells (Treg-type) on CD4 T cells in the liver. (**f**) The percentage of CD8^+^IL-10^+^ T cells (Treg-type) on CD8 T cells in the liver. LD: 50 PSCs; MD: 500 PSCs; HD: 2000 PSCs. Data are shown as mean ± standard error of the mean (SEM, 5–6 mice per group), *p < 0.05, **p < 0.01 and ***p < 0.001.

We investigated T1-type CD4 T cells (CD4^+^IFN-γ^+^, CD4^+^TNF-α^+^) and CD8 T cells (CD8^+^IFN-γ^+^, CD8^+^TNF-α^+^) in the liver. The course of the percentage of these cell subpopulations in each group is shown in Fig. 5 and Supplementary Fig. S4. The percentages of T1-type T cells except for CD8^+^TNF-α^+^ T cells gradually decreased in HDG from 12 to 24 weeks post-infection.

At 2 weeks, the percentage of CD4^+^IFN-γ^+^ T cells was elevated in LDG, MDG and HDG, and there was significantly higher in HDG than in control group (p = 0.0016, control data not shown). The percentage of CD4^+^TNF-α^+^ T cells was lower in MDG and HDG than in LDG (p = 0.0009; p = 0.0038). However, the percentages of CD8^+^IFN-γ^+^ T cells and of CD8^+^TNF-α^+^ T cells were not significantly different among all groups. At 12 weeks, the percentage of CD4^+^IFN-γ^+^ T cells was higher in MDG than in LDG and HDG (p = 0.0344). The percentage of CD8^+^IFN-γ^+^ T cells was higher in HDG than in LDG and MDG (p = 0.0017; p = 0.0292). At 24 weeks, the percentage of CD8^+^IFN-γ^+^ T cells was higher in HDG than in LDG and HDG (p = 0.0003; p = 0.0088). The percentage of CD8^+^TNF-α^+^ T cells was increased in MDG and HDG, but there was no significant difference between all groups.

mRNA expression of T1-type T cell cytokines (IFN-γ, TNF-α, IL-6) was measured in livers from infected mice. At 2 weeks, IFN-γ and IL-6 mRNA expressions were elevated, and IL-6 mRNA expression was higher in HDG than in LDG and MDG. At 12 weeks, IFN-γ mRNA expression was lower in MDG than in LDG. At 24 weeks, IFN-γ, TNF-α and IL-6 mRNA expressions were higher in HDG than in LDG and MDG. (Supplementary Fig. S5).

### Dynamic changes of hepatic T2-type CD4 T cells and related cytokines in mice with different PSC inoculum

We investigated T2-type CD4 T cells (CD4^+^IL-4^+^, Th2) in the liver of infected mice. The course of the percentage of these cell subpopulations in each group is shown in Supplementary Fig. S6.

At 2 weeks, Th2 cell percentage was not significantly different in the 3 groups. At 12 weeks, Th2 cell percentage was higher in MDG than in LDG and HDG (p = 0.0218). At 24 weeks, Th2 cell percentage was higher in HDG than MDG, but there was not statistically different between the 3 groups.

mRNA expression of T2-type T cell cytokines (IL-4, IL-5 and IL-13) was measured in livers from infected mice. At 2 weeks, IL-4, IL-5 and IL-13 mRNA expressions were higher in MDG and HDG than in LDG. At 12 weeks, IL-4, IL-5 and IL-13 mRNA expressions were higher in HDG than in MDG and LDG. At 24 weeks, IL-5 and IL-13 mRNA expressions were still higher in HDG than in LDG and MDG. (Supplementary Fig. S5).

### Dynamic changes of hepatic T17-type CD4 T cells and related cytokines in mice with different PSC inoculum

We investigated T17-type CD4 T cells (CD4^+^IL-17A^+^, Th17) in the liver. The course of the percentage of these cell subpopulations in each group is shown in Supplementary Fig. S6.

At 2 weeks, the percentage of Th17 was higher in MDG than in HDG (p = 0.0046). At 12 and 24 weeks, there was no significant differences in Th17 percentage between the 3 groups.

mRNA expression of T17-type T cell cytokine (IL-17A) was measured in livers from infected mice. At 2 weeks, IL-17A mRNA expression was higher in MDG than in LDG. At 12 weeks, IL-17A mRNA expression was lower in MDG and HDG than in LDG. At 24 weeks, IL-17A mRNA expression was not significantly different in the 3 groups. (Supplementary Fig. S5).

### Dynamic changes of hepatic Treg-type CD4 T cells and CD8 T cells and related cytokines in mice with different PSC inoculum

To explore various aspects of T cell-related tolerance, we investigated Treg-type CD4 T cells (CD4^+^CD25^+^Foxp3^+^, CD4^+^IL-10^+^) and regulatory CD8 T cells (CD8^+^IL-10^+^) in the liver. The course of the percentage of these cell subpopulations in each group is shown in Fig. 5 and Supplementary Fig. S6.

At 2 weeks, CD4^+^CD25^+^Foxp3^+^ T cell percentage was lower in MDG than in LDG (p = 0.0092); CD4^+^IL-10^+^ T cell percentage was higher in MDG and HDG than in LDG; and CD8^+^IL-10^+^ T cell percentage was lower in HDG than in LDG and MDG. At 12 weeks, there were no significant differences in CD4^+^CD25^+^Foxp3^+^ T cells and CD8^+^IL-10^+^ T cell percentages between the 3 groups; however, the percentage of CD4^+^IL-10^+^ T cell was higher in MDG and HDG than in LDG, without significant difference. At 24 weeks, CD4^+^CD25^+^Foxp3^+^ T cell percentage was lower in MDG and HDG than in LDG (p = 0.0086; p = 0.0079). It may be noted that the absolute number of CD4^+^CD25^+^Foxp3^+^ T cells was higher in the livers of HDG than in those of the other groups (data not shown). The percentages of CD4^+^IL-10^+^ T cells and of CD8^+^IL-10^+^ T cells were not significantly different in the 3 groups.

mRNA expression of Treg-type T cell cytokines (IL-10, TGF-β1, FGL-2) was measured in livers from infected mice. At 2 weeks, TGF-β1 and FGL-2 mRNA expressions were elevated in HDG, and TGF-β1 level was higher in HDG than in MDG. At 12 weeks, IL-10, TGF-β1 and FGL-2 mRNA expressions were not significantly different in the 3 groups. At 24 weeks, IL-10, TGF-β1 and FGL-2 mRNA expressions were significantly higher in HDG than in LDG and MDG. (Supplementary Fig. S5).

### Exhaustion markers of T cells in the liver of mice with high dose infection at late stages of infection

We analyzed the inhibitory receptor expression of T cells in the liver of mice with different PSC inoculum to evaluate the level of exhaustion in our model of *E. multilocularis* infection.

**Figure 6:**
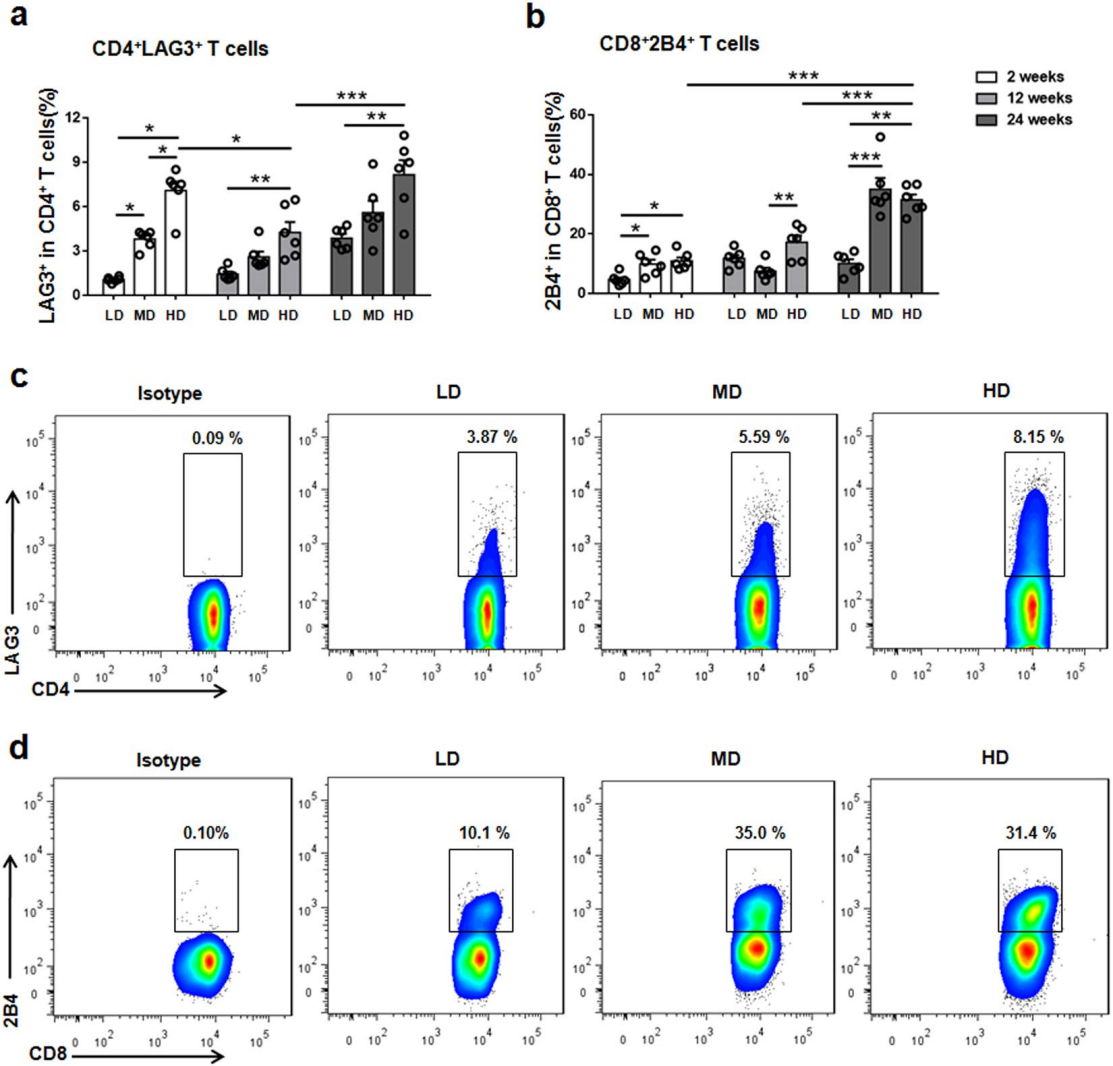
Co-inhibitory receptors LAG3 and 2B4 expression on CD4 T cells and CD8 T cells in mice infected with different *E. multilocularis* PSC inocula during the course of infection. (**a**) The percentage of LAG3 expression on CD4 T cells in the liver. (**b**) The percentage of 2B4 expression on CD8 T cells in the liver. (**c**) Representative FACS plots gated on CD4 T cells showing LAG3 expression in the liver after 24 weeks infection. (**d**) Representative FACS plots gated on CD8 T cells showing 2B4 expression in the liver after 24 weeks infection. LD: 50 PSCs; MD: 500 PSCs; HD: 2000 PSCs. Data are shown as mean ± standard error of the mean (SEM, 5–6 mice per group), *p < 0.05, **p < 0.01 and ***p < 0.001.

At 2 weeks, the percentage of total CD4 T cells expressing LAG3 was higher in MDG and HDG than in LDG. At 12 and 24 weeks, CD4^+^LAG3^+^ T cell percentage was higher in HDG than in LDG (p = 0.0011; p = 0.0013) (Fig. 6a, c). In contrast, there was no significant difference in CD8^+^LAG3^+^ T cell percentages among all groups from 2 to 24 weeks (data not shown).

At 2 weeks, the percentage of CD4 T cells expressing 2B4 was higher in HDG than in LDG and MDG (data not shown); the percentage of CD8 T cells expressing 2B4 was higher in MDG and HDG than in LDG. At 12 weeks, CD8^+^2B4^+^ T cell percentage was higher in HDG than in MDG (p = 0.0045). At 24 weeks, CD8^+^2B4^+^ T cell percentage was significantly higher in HDG and MDG than in LDG (p = 0.0001; p = 0.0002) (Fig. 6b, d).

Based on the above results, we studied the *in vitro* potential of both hepatic CD4 T cells and CD8 T cells from HDG mice to secrete IFN-γ and TNF-α after stimulation with phorbol 12-myristate 13-acetate (PMA)/ Ionomycin. From 12 to 24 weeks post-infection, this potential was actually decreased, indicating that both were undergoing exhaustion at the late stages of infection (Fig. 5a, c).

**Figure 7:**
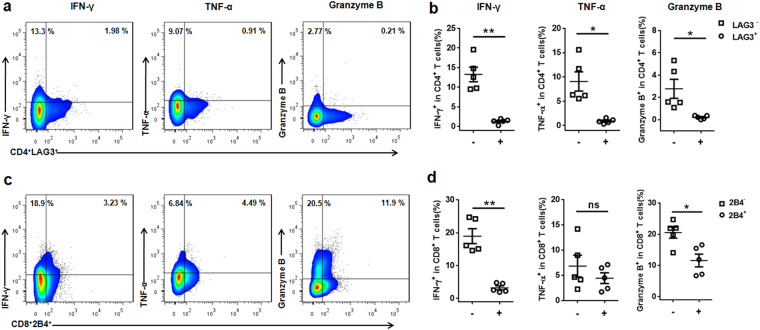
Impaired cytokine responses of LAG3 expressing CD4 T cells and 2B4 expressing CD8 T cells in mice infected with high dose *E. multilocularis* after 24 weeks infection. (**a**) Representative FACS plots gated on CD4 T cells showing LAG3 expression and either IFN-γ, TNF-α, or granzyme B content after stimulation with EmP. (**b**) Compiled data of IFN-γ, TNF-α, or granzyme B CD4 T cell percentage (%) from LAG3^+^ or LAG3^−^ CD4 T cell compartments after EmP stimulation. (**c**) Representative FACS plots gated on CD8 T cells showing 2B4 expression and CD8 T cells showing 2B4 and either IFN-γ, TNF-α, or granzyme B content after stimulation with EmP. (**d**) Compiled data of IFN-γ, TNF-α, or granzyme B CD8 T cell percentage (%) from 2B4^+^ or 2B4^−^ CD8 T cell compartments after EmP stimulation. Data are shown as mean ± standard error of the mean (SEM, 4–5 mice per group), *p < 0.05, **p < 0.01 andns, p > 0.05.

We next assessed the functional status of LAG3 expressing-hepatic CD4 T cells and 2B4 expressing-hepatic CD8 T cells in late-stage infection. Using intracellular cytokine staining, in response to stimulation with EmP or PMA/ Ionomycin, we observed that CD4^+^LAG3^+^ T cells produced significantly less IFN-γ, TNF-α and granzyme B than CD4^+^LAG3^−^ T cells. Moreover, CD8^+^2B4^+^ T cells produced significantly less IFN-γ, TNF-α and granzyme B than CD8^+^2B4^−^ T cells. (Fig. 7a–d and Supplementary Fig. S 7).

## Discussion

The common immunological knowledge of experimental *E. multilocularis* infection comes from studies using experimental mice with intraperitoneal, subcutaneous or intrahepatic infection^18–20^. Although portal vein injection might be more relevant, in terms of location of secondary *E. multilocularis* infection, there are very few reports on the portal route to inoculate *E. multilocularis* metacestode into intermediate hosts^21^, and they have not addressed the immunological components of *E. multilocularis* infection. In the present study, we developed a suitable experimental model that mimics naturally infected livers to comprehensively study the outcome of infection and the differential regulation of the immune response according to the parasite load. Depending on the number of PSCs injected intraportally, this model reliably reproduces the establishment of the metacestode and pathological changes observed in resistant humans (LDG), in humans with AE (MDG), or in susceptible natural or experimental rodent hosts (HDG). The immune profiles in terms of cell subpopulations and cytokines also globally fit with the descriptions previously made in humans and experimental mice; in addition, we provide additional data that comprehensively draw a more complete picture of the balance between protection and tolerance immunity at the various stages of the disease (Supplementary Fig. S8), and suggest that T cell exhaustion takes part in the overall enhancement of metacestode growth at the late stages of infection.

Macroscopic observations of the livers from different dose groups in our model are similar to those of the primary infection model at 2 weeks^7^. In addition, PSC injection provides more constant and reliable results than primary infection with eggs: of the 8 C57/B6 mice with high dose infection examined at 16 weeks post-infection, 7 had echinococcal foci or vesicles, which were observable separately at the surface of the liver lobes, and had a lesion distribution similar to that observed in primary infection^7^. This confirmed the initial observations made by Nakaya *et al*.^22^ who also stressed that this ‘trans-portal’ model, in rats or in mice, allowed a strict location of lesions to the liver. In addition, in our mouse model using PSCs, we were able to control the precise parasite load; this is an important point, since imprecision on parasite load when metacestode homogenate is used for secondary infection makes a complex design of experiments necessary to ensure absence of bias in the parasite load of all mice groups in experimental studies on *E. multilocularis*. Liver location of AE lesions is important when the aim of the experimental model is to study the immune response. The liver is a key organ that controls the metabolic homeostasis of lipids, carbohydrates, and proteins. Accumulative evidence demonstrates that the liver has specific immunological properties and contains a large number of resident and non-resident cells that participate in the regulation of inflammatory and immune responses, and maintains hepatic and systemic homeostasis^23^. Effective local immunity is essential for detecting and clearing many hepatotropic pathogens, including several species of viruses, bacteria and parasites^24^. However, dysregulation of liver non-specific inflammatory response is a hallmark of chronic infection^25^. Persistent activation of immune response leads to progressive liver fibrosis and permanent liver damage^26^. In these situations, chronic pathological inflammation promotes the progression of liver fibrosis to cirrhosis and establishes a dysregulated balance between inflammation and immunosuppression within the liver^27^.

Our results demonstrate that at the early stage of infection (2 weeks), all LD or MD mice showed minute foci in the liver caused by the parasite, thus were actually infected; then at the middle and later stage (from 4 to 24 weeks), the foci almost disappeared at the surface of livers and most of them healed. Our pathological observations confirm that local cellular immunity and fibrogenesis are actually protective and fully able to limit metacestode growth in the liver of LD or MD mice^3, 28^, or even to clear it, as was the case in most of animals of these groups, especially in the LDG, which appears to be a good model of ‘aborted’ or ‘died-out’ AE. In HDG, pathological observations suggest that impairment of cellular immunity is followed by a more rapid and severe course of the disease. Like previous sequential studies on AE^29, 30^, our results showed that the absolute numbers of NK, T cells and B cells were significantly increased in the livers of HDG mice. In addition, higher numbers of hepatic CD4 T cells and CD8 T cells were also found in the HDG, and there was a dramatic decrease of the CD4/CD8 ratio. Similar studies in AE patients have also shown that the relative ratio of T cell subsets in the liver affects the efficiency of the granuloma and thus the outcome of infection^2^. Furthermore, we tested the phenotypic characteristic of the responding T cells population in the liver of mice with different dose infection. In studies of patients with AE, thus long after the initiation of the disease, the generation of memory Th1 CD4 T cells was shown to be impaired^31^. Our data showed that the percentage of CD4 Tem cells was significantly increased in HDG at the late stage and CD8 Tem cells were also present in the HDG livers. However, the percentage of CD4 Tcm cells was very low in the liver of infected mice, but a higher percentage of CD8 Tcm cells were found in the liver of mice with *E. multilocularis* infection. These results suggest that *E. multilocularis* infection can result in a fast transition to CD8 T cell memory responses, which develop as early as 2 weeks. Moreover, CD8 Tcm cells can be maintained once parasites are no longer present in the liver, as was observed in MDG at 24 weeks post-infection. Our findings are in agreement with previous studies on infectious agents that, like *E. multilocularis*, lead to granuloma formation in the liver, e.g. *Leishmania*, *Mycobacterium* and *Schistosoma* spp; in these models, it was shown that the hepatic granulomas had memory lymphocytes with down-regulated CD62L levels as the main infiltrating cells^32–34^. Taken together, these results suggest that recruitment and/ or proliferation of memory T cells in liver was not only associated with clearance of the parasite infection in the LDG, but also with increased hepatic injury in the HDG, which might be due to the involvement of regulatory T cells or related cytokines in suppressing “protective” immune responses in the liver of mice with chronic infection. Recent studies have shown that memory T cells are heterogeneous; containing subsets that can become Th1, Th2 or other T cell types^35, 36^.

Immune-mediated liver injury is actually an important aspect of AE pathogenesis. The host immune response to *E. multilocularis* infection has been shown to be a T cell-dependent process, which involves all types of T cell-related responses^37–39^. Sequential and combined T1, T2, T17-type T cells and Treg profiles appear crucial for the generation of either effective or ineffective response resulting in self-healing or in the maintenance of a chronic infection, thus determining the prognosis of *E. multilocularis* infection^3, 40, 41^. T1-type and T17-type T cells can release pro-inflammatory cytokines, which have been implicated in host defense against a number of infectious organisms^42^, and participate in liver tissue immune cell infiltration and in fibrogenesis during *E. multilocularis* infection^43^. By contrast, T2-type T cells and various regulatory T cells display suppressive and surveillance functions in immune responses and inflammatory diseases^44^. They can build a micro-environment with immuno-suppressive function through the secretion of cytokines such as IL-4, IL-5, IL-10, IL-13, and TGF-β1, and regulatory proteins such as FGL-2, to inhibit the maturation of antigen-presenting cells or endow T cells with immunoregulatory phenotypes, which is usually called ‘infectious tolerance’^45, 46^. Tolerance phenomena, which involved all the above mentioned components, have been well shown in patients with AE and consistently demonstrated in experimental models of *E. multilocularis* infection^3^. Our data fully support their conclusions, which make our model valid regarding the main events of the immune response, and in addition show how the parasite load at the initiation of infection may modulate these various components to lead to opposite outcomes, from complete clearance to fast and extensive growth of the metacestode. Our data on TNF-α- and INF-γ- producing CD8 T cells, and on IL-10- producing CD4 and CD8 T cells, never studied in previous reports, are particularly interesting to this regard; in particular they well showed the dual role of CD8 T cells depending on the parasite load and at the various stages of metacestode growth. CD8 T cells are well known to represent the major component of the hepatic periparasitic infiltrate in progressive *E. multilocularis* infection but elucidation of their role is only at the beginning^2^. Our data on their cytokine production and on their markers of exhaustion provide new insights into this role.

**Figure 8:**
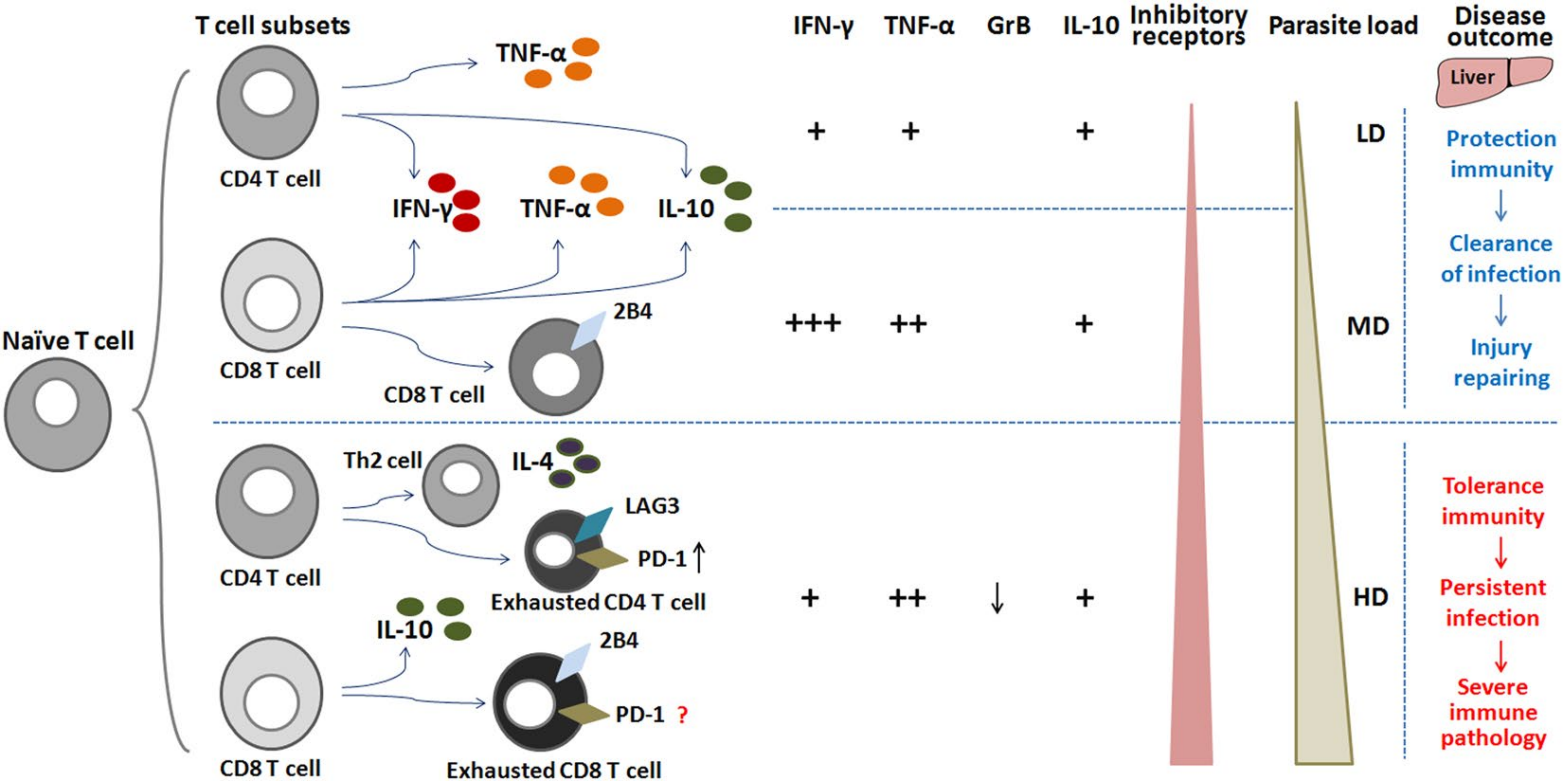
The balance between T cell response and parasite load determines the disease outcome of mice infected with different *E. multilocularis* PSC inocula. Hepatic naïve T cells (Tn) are primed and differentiate into different T cell subsets, including T1-type, T2-type, T17-type and Treg-type T cells from mice infected with different *E. multilocularis* PSC inocula. A low or medium parasitic load result in a protective T cell response that limits metacestode growth or even clears it, and repairs liver injury. A high parasitic load is associated with tolerance to the metacestode which counterbalances the protective response. In addition, in situations of repeated antigen stimulation due to high parasitic load and persistent inflammation, effector T cells (Te) express multiple inhibitory receptors (e.g. LAG3, 2B4, PD-1^53^) and gradually lose their capacity to exert cytotoxicity and to produce cytokines, which enhances metacestode growth. LD: 50 PSCs; MD: 500 PSCs; HD: 2000 PSCs.

In susceptible mice, the initial slow development of the metacestode, followed by a rapid rise in lesion size at the middle/late stages of infection, associated with a global decrease in lymphocyte functions, has been known for decades, and constantly confirmed^18, 40^. In patients with AE, absence of expression of the co-stimulatory receptor NKG2D at the surface of CD8 T cells in the periparasitic granuloma, despite elevated expression of its ligands, MICA and MICB, and thus inhibition of their cytotoxicity functions, has been reported^36^. We hypothesized that the phenomenon commonly called ‘T cell exhaustion’ could play a role in these observations and add to the well-described immune tolerance process in intermediate hosts with chronic infection. Positive expression of inhibitory receptors such as programmed death receptor (PD-1), LAG3 and 2B4 has been directly linked to reduced cytokine production of T cells from cancer patients or in the chronic viral mouse model of ‘T cell exhaustion’ and other parasitic infections^14, 16, 44^. Our results fully confirm this hypothesis. We could show that there were increased percentages of LAG3 expressing CD4 T cells and of 2B4 expressing CD8 T cells in mice with high parasite load during chronic infection. And we were also able to demonstrate *in vitro* that hepatic CD4^+^LAG3^+^ T cells and CD8^+^2B4^+^ T cells actually displayed impaired effector phenotype, indicating altered functions of both subpopulations of T cells in mice which could not clear infection because of high PSC inoculum (Fig. 8). T cell exhaustion has been suggested to be a mechanism for the pathogen to survive by not killing its host; however, it may also be a mechanism for the host to survive high dose of pathogens whether it is from a high initial inoculum or from pathogens that grow/replicate fast. For example, during infection with a more virulent *P. yoelii* variant, CTLA-4 blockade induced markedly increased serum levels of TNF-α accompanied by severe inflammation and reduced survival^47^. During *Trypanosoma cruzi* infection, CTLA-4 blockade resulted in increased NO production *in vivo* and *in vitro*, and increased resistance to infection with the Y and Colombian strains of *T. cruzi*, and also led to increased survival rate^48^. The reality of the phenomenon is now demonstrated in *E. multilocularis* infection; however, detailed mechanisms associated with LAG3 and 2B4 expression, and other markers of T cell exhaustion, during chronic infection in mice with high parasitic load, and their consequences on the host, certainly deserve further studies.

## Methods

### Ethics statement

All animals received humane care in compliance with the Medical Research Center’s guidelines, and animal procedures were approved by the Animal Care and Use Committee and the Ethical Committee of First Affiliated Hospital of Xinjiang Medical University (20140411-05). All surgery was performed under chloral hydrate anesthesia, and all efforts were made to minimize suffering.

### Animals and procedures

Pathogen-free female C57BL/6 mice (8–10 weeks old) were purchased from the Beijing Vital river Experimental Animal Technology Co. Ltd., and were maintained in an air-conditioned animal room with a 12-h light/dark cycle and provided with rodent chow and water. *E. multilocularis* PSCs were obtained from intraperitoneal lesions maintained in BALB/c. Briefly, the parasites were washed several times using phosphate buffered saline (PBS), pH = 7.2, containing 1000 mg/mL penicillin and 1000 U/mL streptomycin (Hyclone, Beijing, China), counted using a DMI 4000B microscope (Leica, Germany) and adjusted to the appropriate parasite concentration before injection. The parasite vitality was determined by eosin exclusion^49^. Only parasite batches exhibiting over 95% vitality were used. Mice were inoculated via the hepatic portal vein with different doses live PSCs in saline, whereas control mice were injected with isotonic saline. Mice were anesthetized, and an incision was made in the skin and peritoneum of the middle of the body. All mice received 200–300 μL of PSCs sediment via the hepatic portal vein by using a 0.45 × 15RWLB venous infusion needle. Immediately after injection, light compression to the puncture site was performed using a cotton bud for 3 min to provide haemostasis and to prevent intraperitoneal spillage of the PSCs. Mice were sacrificed at 2, 4, 8, 12, 16, 20 and 24 weeks. The surface of livers was carefully screened for hepatic lesions for a preliminary assessment of the intensity of *E. multilocularis* infection at different times.

### *E. multilocularis* antigen preparation

For *E. multilocularis* PSCs antigen (EmP) preparation, entire *E. multilocularis* PSCs were collected from intraperitoneal lesions maintained in BALB/c, and PSCs were ruptured by sonication pulses (30% intensity, pulse 1 s for 1 min). Such disrupted PSCs were then homogenized, that is, grinded in liquid nitrogen until a homogenous liquid extract was produced, and then thereafter the PSCs homogenate was centrifuged at 12000 g for 30 min at 4 °C. The supernatant was sterilely filtrated (0.45 μm) and stored at −70 °C until use.

### Tissue sampling and histopathological analysis

For liver histopathology, all liver lobes were separated and placed in 10% buffered formalin and then embedded in paraffin. Paraffin-embedded 5 μm sections were mounted on glass slides and stained with the Hematoxylin-Eosin to evaluate infiltrating cells and granuloma formation. Liver histological reactions surrounding the infectious foci were classified into three categories (a) ‘fibrotic foci’: parasite-free, no visible PSCs or metacestode, only liver fibrosis; (b) ‘inflammatory foci with fibrosis’: parasite-free, no visible PSCs or metacestode, small inflammatory foci with liver fibrosis; (c) ‘infectious foci with metacestode structure’: fibroblasts, myofibroblast, lymphocytes and monocytes surrounding parasitic lesion with identified germinal layer. This result was expressed as percentage of each type of infectious focus to the number of all parasitic foci. The lesion areas in 10–15 fields/section/mouse ( × 100) were measured by computer-assisted morphometric analysis using cellSens Dimension software (Olympus, Tokyo, Japan) and expressed as square micrometers (μm^2^).

For liver fibrosis, paraffin-embedded 5 μm sections were stained with the picric acid-Sirius red technique to evaluate collagen fibers, as described previously^50^. This was assessed at × 100 magnifications in a total of 10–15 fields/section/ mouse, using computerized quantification and results were expressed as percentage of picric acid-sirius red staining per field.

For qRT-PCR analysis, liver tissue samples were also taken from the parasitic lesion (including periparasitic liver tissue adjacent by 1–2 mm to the macroscopically visible parasitic lesion) in *E. multilocularis* infected mice. In control mice, liver tissue samples were taken from the same (anterior) liver lobe in the sham-operated area. Tissue fragments were directly deep-frozen in liquid nitrogen.

### Immunohistochemistry analysis

For IHC studies, paraffin-embedded liver tissue samples of control mice or infected mice were examined to determine the expression and distribution of α-smooth muscle actin (α-SMA antibody, dilution 1:1000, Abcam, UK) protein at each time points, as described elsewhere^17^. Sections were examined microscopically for specific staining and photographs were taken using a digital image-capture system (Olympus, Tokyo, Japan); the intensity of positive staining was quantified histologically using computer-assisted morphometric analysis using cellSens Dimension software (Olympus,, Tokyo, Japan)^51^.

### Liver mononuclear cell isolation and flow cytometry analysis

Leukocytes were isolated from liver as described previously^52^. For flow cytometry, cell preparations were incubated with purified anti-CD16/CD32 antibody (BioLegend, San Diego, CA) for 15 min at 4 °C, and then stained with the following antibodies conjugated to fluorescent labels: anti-NK1.1, anti-CD3, anti-CD4, anti-CD8β, anti-CD19, anti-CD44, anti-CD62L, LAG3 and 2B4 (BioLegend).

For intracellular cytokine staining, approximately 10^6^ liver mononuclear cells were stimulated with Cell Stimulation Cocktail (plus protein transport inhibitors) (eBioscience, California, USA) for 4 h, or EmP (5 μg/ml) for 10 h (37 °C, 5% CO2) as previously described^52^. The cells were stained for surface anti-NK1.1, anti-CD3, anti-CD4, anti-CD8β, LAG3 and 2B4, and then stained for intracellular anti-IFN-γ, anti-IL-4, anti-IL-17A, anti-TNF-α, anti-IL-10 and granzyme B (Biolegend). IgG isotype controls (Biolegend) were used in parallel. To detect Treg cells, cells were stained with anti-NK1.1, anti-CD3, anti-CD4, anti-CD25 at 4 °C for 30 minutes; and incubated with anti-mouse Foxp3 antibodies according to the manufacturer’s instructions (eBioscience). ALSRFortessa flow cytometry (BD Immunocytometry Systems, San Jose, CA) was used to acquire data, which were analyzed with Flowjo software (Treestar, Inc., Ashland, OR).

### Quantitative real-time PCR analysis

Total RNA was extracted from liver tissues using Trizol Reagent (Invitrogen, Carlsbad, CA). M-MLV Transcriptase was used for reverse transcription according to the manufacturer’s instructions (Thermo, Waltham, USA). The qRT-PCR was run in a thermocycler (iQ5 Bio-Rad, Hercules, CA) with the SYBR Green PCR premix (TaKaRa, Dalian, China) as described previously^17^. The PCR primers used herein are noted in Table 1. The results were calculated by the 2^−△△Ct^ method.

**Table 1:** Sequences of the qRT-PCR primers.

Gene	Genbank accession	Forward primer	Reverse primer
Acta2	NM_007392.3	AAGAGCATCCGACACTGCTGAC	AGCACAGCCTGAATAGCCACATAC
COL1A1	NM_007742.4	CAGGGTATTGCTGGACAACGTG	GGACCTTGTTTGCCAGGTTCA
IFN-γ	NM_008337.4	TAGCCAAGACTGTGATTGCGG	AGACATCTCCTCCCATCAGCAG
TNF-α	NM_013963.3	AAGCCTGTAGCCCACGTCGTA	AGGTACAACCCATCGGCTGG
IL-6	NM_031168.2	CCACTTCACAAGTCGGAGGCTTA	CCAGTTTGGTAGCATCCATCATTTC
IL-4	NM_021283.2	ACAGGAGAAGGGACGCCAT	GAAGCCCTACAGACGAGCTCA
IL-5	NM_010558.1	TGGGGGTACTGTGGAAATGC	CCACACTTCTCTTTTTGGCGG
IL-10	NM_010548.2	GCCAGAGCCACATGCTCCTA	GATAAGGCTTGGCAACCCAAGTAA
IL-13	NM_008355.3	CCCTGGATTCCCTGACCAAC	GGAGGCTGGAGACCGTAGT
IL-17A	NM_010552.3	ACCGCAATGAAGACCCTGAT	TCCCTCCGCATTGACACA
TGF-β1	NM_011577.2	GTGTGGAGCAACATGTGGAACTCTA	CGCTGAATCGAAAGCCCTGTA
FGL2	NM_008013.4	TGGACAACAAAGTGGCAAATCT	TGGAACACTTGCCATCCAAA
GAPDH	NM_001289726.1	CATGGCCTTCCGTGTTCCTA	CCTGCTTCACCACCTTCTTGAT

## Statistical analysis

Statistical significance was analyzed using GraphPad Prism 6.0 (GraphPad Software, San Diego, CA). Results are expressed as mean ± standard error of the mean. The one-way ANOVA test with a Tukey’s multiple comparison was used when there were more than two groups. The Student’s t -test was used when only comparing two groups. Critical for significant differences was a p-value less than 0.05 for all experiments. (p-values were expressed as follows: *p values < 0.05; **p values < 0.01; ***p values < 0.001).

## Electronic supplementary material

Supplementary info
