# Author Correction: T-cell tolerance and exhaustion in the clearance of *Echinococcus multilocularis*: role of inoculum size in a quantitative hepatic experimental model

**DOI:** 10.1038/s41598-019-39975-9

**Published:** 2019-02-27

**Authors:** Chuanshan Zhang, Yingmei Shao, Shuting Yang, Xiaojuan Bi, Liang Li, Hui Wang, Ning Yang, Zhide Li, Cheng Sun, Liang Li, Guodong Lü, Tuerganaili Aji, Dominique A. Vuitton, Renyong Lin, Hao Wen

**Affiliations:** 1 State Key Laboratory Incubation Base of Xinjiang Major Diseases Research, and WHO Collaborating Centre on Prevention and Case Management of Echinococcosis, Clinical Medicine Institute, The First Affiliated Hospital of Xinjiang Medical University, Urumqi, Xinjiang China; 2 Department of Hepatic Hydatid and Hepatobiliary Surgery, Digestive and Vascular Surgery Centre, The First Affiliated Hospital of Xinjiang Medical University, Urumqi, Xinjiang China; 3 Xinjiang Key Laboratory of Echinococcosis, Clinical Medicine Institute, The First Affiliated Hospital of Xinjiang Medical University, Urumqi, Xinjiang China; 4 Institute of Immunology, The Key Laboratory of Innate Immunity and Chronic Disease (Chinese Academy of Medical Science), School of Life Sciences and Medical Center, University of Science & Technology of China, Hefei, Anhui China; 5 WHO-Collaborating Centre for the Prevention and Treatment of Human Echinococcosis, Department of Parasitology, University Bourgogne Franche-Comté (EA 3181) and University Hospital, Besançon, France

Correction to: *Scientific Reports* 10.1038/s41598-017-11703-1, published online 11 September 2017

This Article contains typographical errors in the Acknowledgements section.

“This work was supported by Xinjiang Uygur Autonomous Region Science and Technology Program (No. 201430123) and National Natural Science Foundation of China (U1303222, 81660341, 81560330, 81560098).”

should read:

“This work was supported by Xinjiang Uygur Autonomous Region Science and Technology Program (No. 201430123) and National Natural Science Foundation of China (U1303222, 81371838, 81560330, 81560098).”

