# Supplementary material for: T-cell tolerance and exhaustion in the clearance of Echinococcus multilocularis: role of inoculum size in a quantitative hepatic experimental model
Source: Sci Rep. 2017 Sep 11;7:11153. doi: 10.1038/s41598-017-11703-1 (PMC5593833; doi:10.1038/s41598-017-11703-1)

**T-cell tolerance and exhaustion in the clearance of *Echinococcus multilocularis*: role of inoculum size in a quantitative hepatic experimental model**

Chuanshan Zhang^1, #^, Yingmei Shao^2, #^, Shuting Yang^1, #^, Xiaojuan Bi^1^, Liang Li^4^, Hui Wang^3^, Ning Yang^1^, Zhide Li^1^, Cheng Sun^4^, Liang Li^3^, Guodong Lü^1^, Tuerganaili Aji^2^, , Dominique A. Vuitton^5^, Renyong Lin^1^, *, Hao Wen^1, 2^, *.

1. State Key Laboratory Incubation Base of Xinjiang Major Diseases Research, and WHO Collaborating Centre on Prevention and Case Management of Echinococcosis, Clinical Medicine Institute, The First Affiliated Hospital of Xinjiang Medical University, Urumqi, Xinjiang, China.

2. Department of Hepatic Hydatid and Hepatobiliary Surgery, Digestive and Vascular Surgery Centre, The First Affiliated Hospital of Xinjiang Medical University, Urumqi, Xinjiang, China

3. Xinjiang Key Laboratory of Echinococcosis, Clinical Medicine Institute, The First Affiliated Hospital of Xinjiang Medical University, Urumqi, Xinjiang, China.

4. Institute of Immunology, The Key Laboratory of Innate Immunity and Chronic Disease (Chinese Academy of Medical Science), School of Life Sciences and Medical Center, University of Science & Technology of China, Hefei, Anhui, China

5. WHO-Collaborating Centre for the Prevention and Treatment of Human Echinococcosis, Department of Parasitology, University Bourgogne Franche-Comté (EA 3181) and University Hospital, Besançon, France.

*Corresponding author: Renyong Lin, renyong_lin@sina.com; Hao Wen, Dr.wenhao@163.com

^#^Contributing equally

**Supplementary Figure 1. Macroscopic views of the liver in mice infected with different *E. multilocularis* PSC inocula during the course of infection.** The whitish dots on the livers represent established parasites. PSCs: protoscoleces. Yellow circle highlight metacestode tissue in the liver. Representative samples are presented.

**Supplementary Figure 2. Hepatic fibrosis in mice infected with different *E. multilocularis* PSC inocula during the course of infection.** (a) Liver fibrosis as determined by picric acid-Sirius red staining (original magnification×100). The red area represents fibrillar collagen. (b) The fibrosis area of the section was quantified using cellSens Dimension software, the ratio of collagen area and total area (%) was counted. (c) mRNAs of COL1A1 in liver total RNA were quantified by qRT-PCR. (d) Immunohistochemical staining for α-SMA for detection of activated hepatic stellate cells (HSCs). (e) The percentage of positive staining cells was calculated to assess the expression of α-SMA on the lesion areas at 2, 12 and 24 weeks post-infection. (f) mRNAs of α-SMA (Acta2) in liver total RNA were quantified by qRT-PCR. Bars indicate 200 μm or 50 μm in the 100× or in the 400× magnification images, respectively. C: control; LD: 50 PSCs; MD: 500 PSCs; HD: 2000 PSCs. Data are shown as mean ± standard error of the mean (SEM, 5–6 mice per group), *p < 0.05, **p < 0.01 and ***p < 0.001.

**Supplementary Figure 3. Hepatic memory T cell phenotypes in mice infected with different *E. multilocularis* PSC inocula after 24 weeks infection.** Expression of CD44 and CD62L in hepatic CD4 T and CD8 T cells from mice with different PSC inocula. Representative FACS plots are shown. LD: 50 PSCs; MD: 500 PSCs; HD: 2000 PSCs. Data are shown as mean values (5–6 mice per group).

**Supplementary Figure 4. Representative FACS plots gated on hepatic T cell subsets in mice infected with different *E. multilocularis* PSC inocula after 24 weeks infection.** (a) Intracellular staining of IFN-γ^+^ in hepatic CD4 T cells from mice with different PSC inocula. (b) Intracellular staining of TNF-α^+^ in hepatic CD4 T cells. (c) Intracellular staining of IFN-γ^+^ in hepatic CD8 T cells. (d) Intracellular staining of TNF-α^+^ in hepatic CD8 T cells. (e) Intracellular staining of Foxp3^+^ in hepatic CD4^+^ CD25^+^ T cells. (f) Intracellular staining of IL-10^+^ in hepatic CD8 T cells. Representative FACS plots are shown. LD: 50 PSCs; MD: 500 PSCs; HD: 2000 PSCs.

**Supplementary Figure 5. Kinetics of hepatic cytokine gene expression in mice infected with different *E. multilocularis* PSC inocula during the course of infection.** Quantitative RT-PCR analysis for mRNA levels of cytokines in whole-liver tissue at various time points after infection and normalized by comparison to the housekeeping gene GAPDH mRNA. (a) IFN-γ. (b) TNF-α. (c) IL-6. (d) IL-4. (e) IL-5. (f) IL-13. (g) IL-17A. (h) IL-10. (i) TGF-β1. (j) FGL-2. LD: 50 PSCs; MD: 500 PSCs; HD: 2000 PSCs. Data are shown as mean ± standard error of the mean (SEM, 5–6 mice per group), *p < 0.05, **p < 0.01 and ***p < 0.001.

**Supplementary Figure 6.** **Distribution of hepatic T cell subsets in mice infected with different *E. multilocularis* PSC inocula during the course of infection.** (a) The percentage of CD4^+^IL-4^+^ T cells (T2-type) on CD4 T cells in the liver. (b) The percentage of CD4^+^IL-17A^+^ T cells (T17-type) on CD4 T cells in the liver. (c) The percentage of CD4^+^IL-10^+^ T cells (Treg-type) on CD4 T cells in the liver. Representative FACS plots are shown (d-f): (d) Intracellular staining of IL-4^+^ in hepatic CD4 T cells after 24 weeks infection. (e) Intracellular staining of IL-17A^+^ in hepatic CD4 T cells after 24 weeks infection. (f) Intracellular staining of IL-10^+^ in hepatic CD4 T cells after 24 weeks infection. LD: 50 PSCs; MD: 500 PSCs; HD: 2000 PSCs. Data are shown as mean ± standard error of the mean (SEM, 5–6 mice per group), *p < 0.05, **p < 0.01 and ***p < 0.001.

**Supplementary Figure 7. Impaired cytokine responses of LAG3 expressing CD4 T cells and 2B4 expressing CD8 T cells in mice infected with high dose *E. multilocularis* after 24 weeks infection.** (a) Representative FACS plots gated on CD4 T cells showing LAG3 expression and either IFN-γ, TNF-α, or granzyme B content after stimulation with a positive control stimulation with PMA/Ionomycin. (b) Compiled data of IFN-γ, TNF-α, or granzyme B CD4 T cell percentage (%) from LAG3^+^ or LAG3^-^ CD4 T cell compartments after PMA/Ionomycin stimulation. (c) Representative FACS plots gated on CD8 T cells showing 2B4 expression and CD8 T cells showing 2B4 and either IFN-γ, TNF-α, or granzyme B content after stimulation with PMA/Ionomycin. (d) Compiled data of IFN-γ, TNF-α, or granzyme B CD8 T cell percentage (%) from 2B4^+^ or 2B4^-^ CD8 T cell compartments after PMA/Ionomycin stimulation. Data are shown as mean ± standard error of the mean (SEM, 4–5 mice per group), *p < 0.05, **p < 0.01 and ***p < 0.001.

**Supplementary Figure 8. Overall changes of T cell subsets and related cytokine profiles in the liver from mice infected with different *E. multilocularis* PSC inocula during the course of infection.**

**Supplementary Figure 1. Macroscopic views of the liver in mice infected with different *E. multilocularis* PSC inocula during the course of infection.**

**
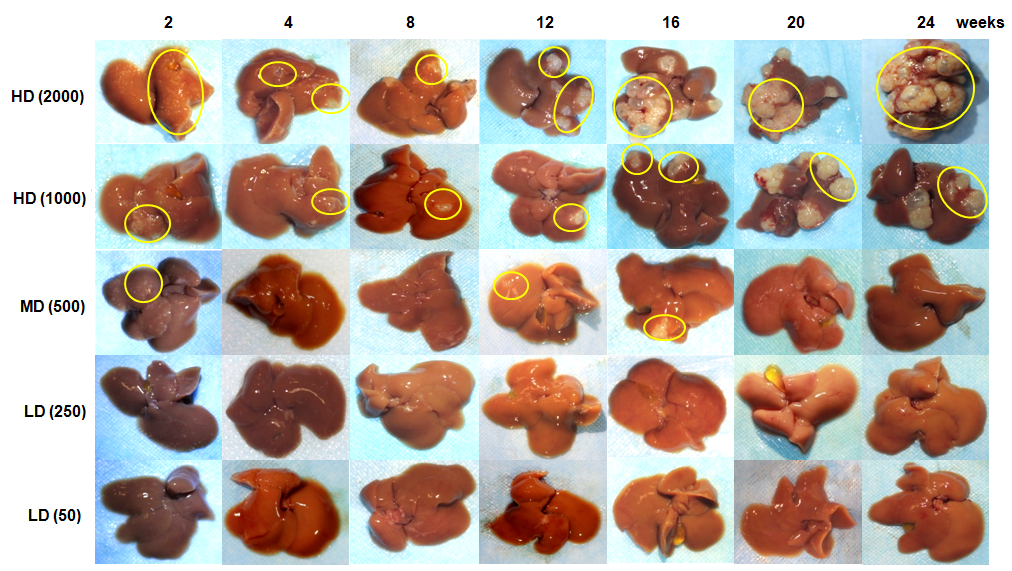
**

**Supplementary Figure 2. Hepatic fibrosis in mice infected with different *E. multilocularis* PSC inocula during the course of infection.**


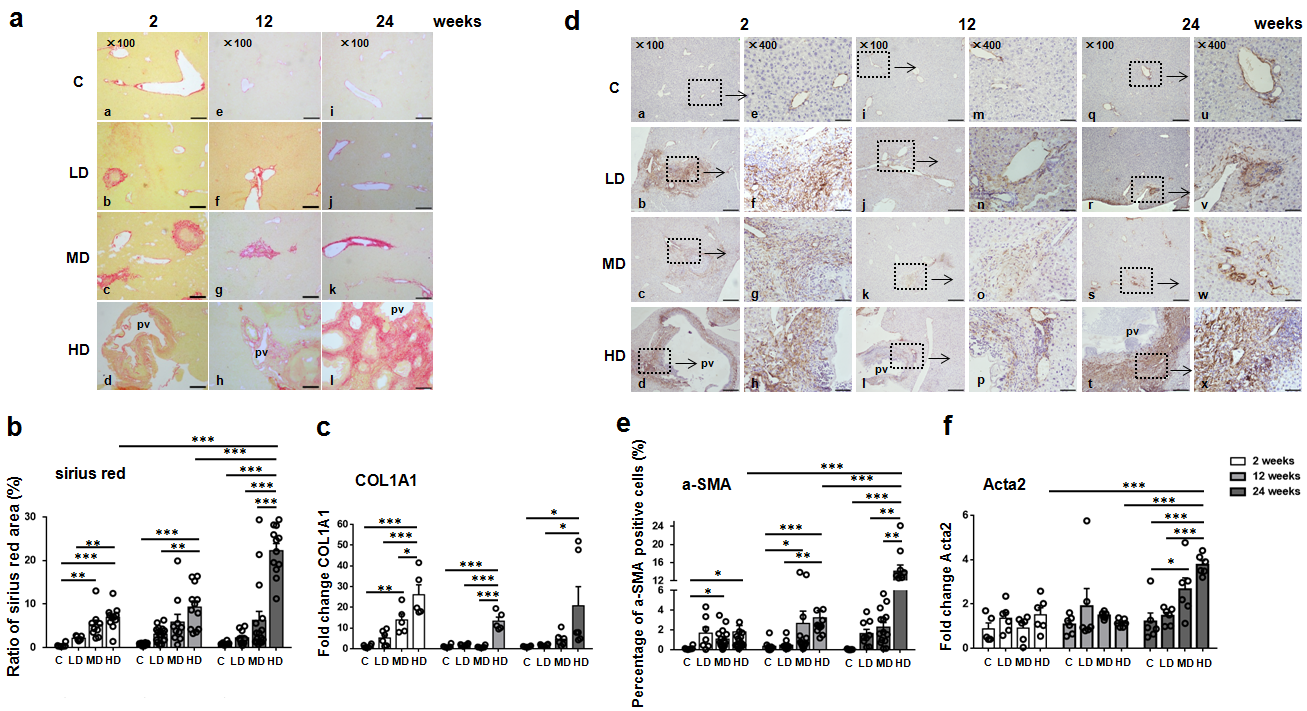


**Supplementary Figure 3. Hepatic memory T cells phenotype in mice infected with different *E. multilocularis* PSC inocula after 24 weeks infection.**


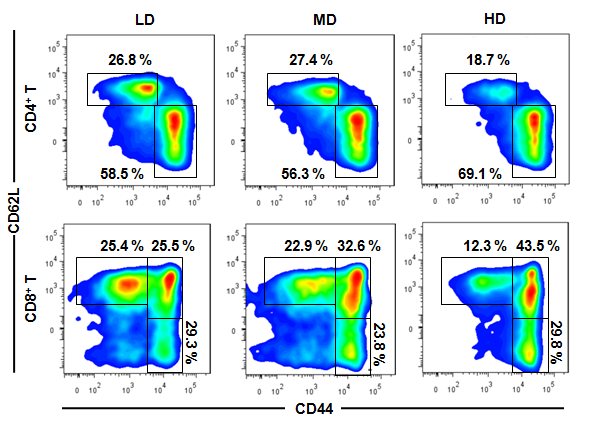


**Supplementary Figure 4. Representative FACS plots gated on hepatic T cell subsets in mice infected with different *E. multilocularis* PSC inocula after 24 weeks infection.**


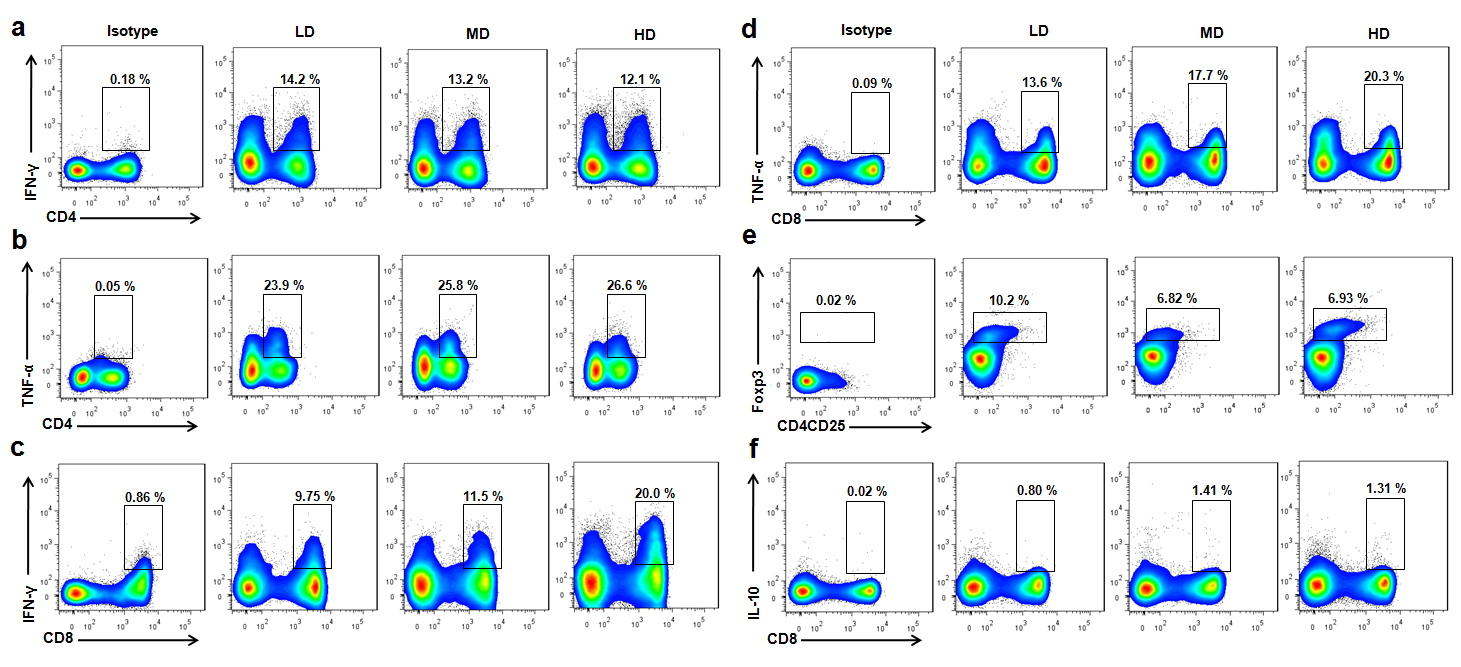


**Supplementary Figure 5. Kinetics of hepatic cytokine gene expressions in mice infected with different *E. multilocularis* PSC inocula during the course of infection.**


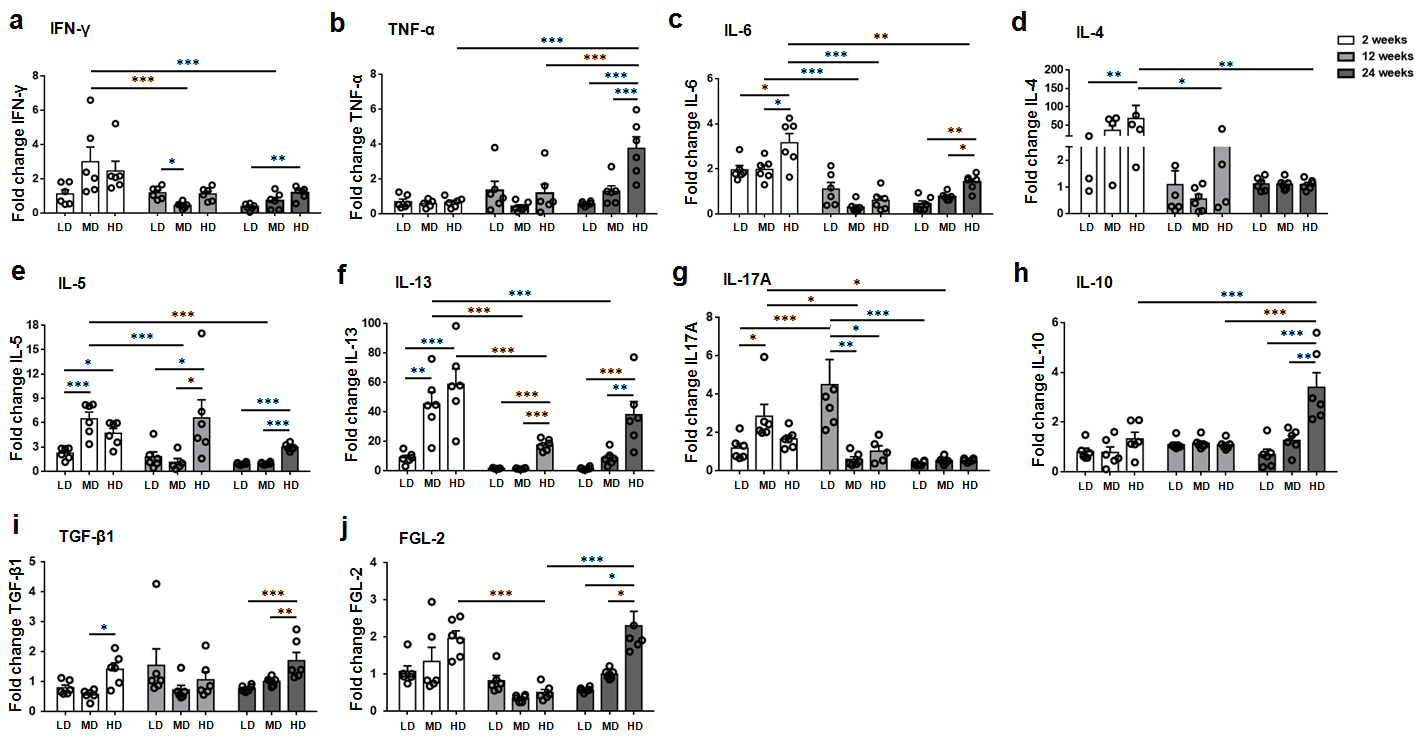


**Supplementary Figure 6. Distribution of hepatic T cell subsets in mice infected with different *E. multilocularis* PSC inocula during the course of infection.**

**
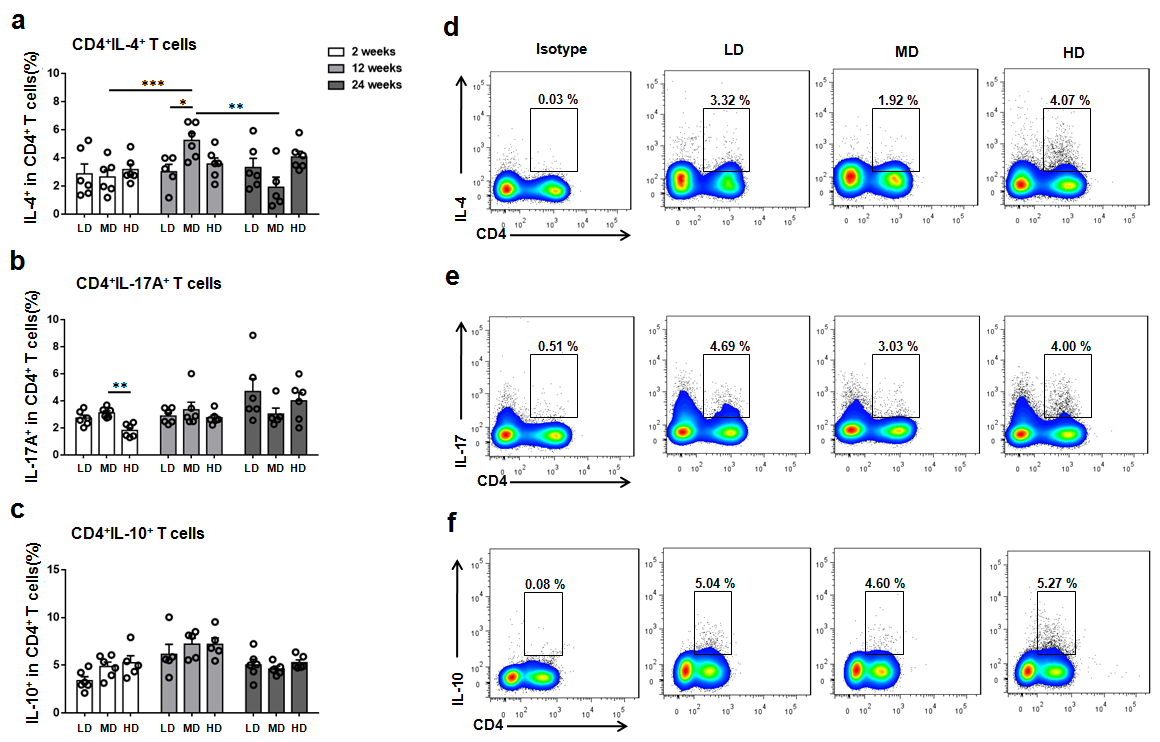
**

**Supplementary Figure 7. Impaired cytokine responses of LAG3 expressing CD4 T cells and 2B4 expressing CD8 T cells in mice infected with high dose *E. multilocularis* after 24 weeks infection.**

**
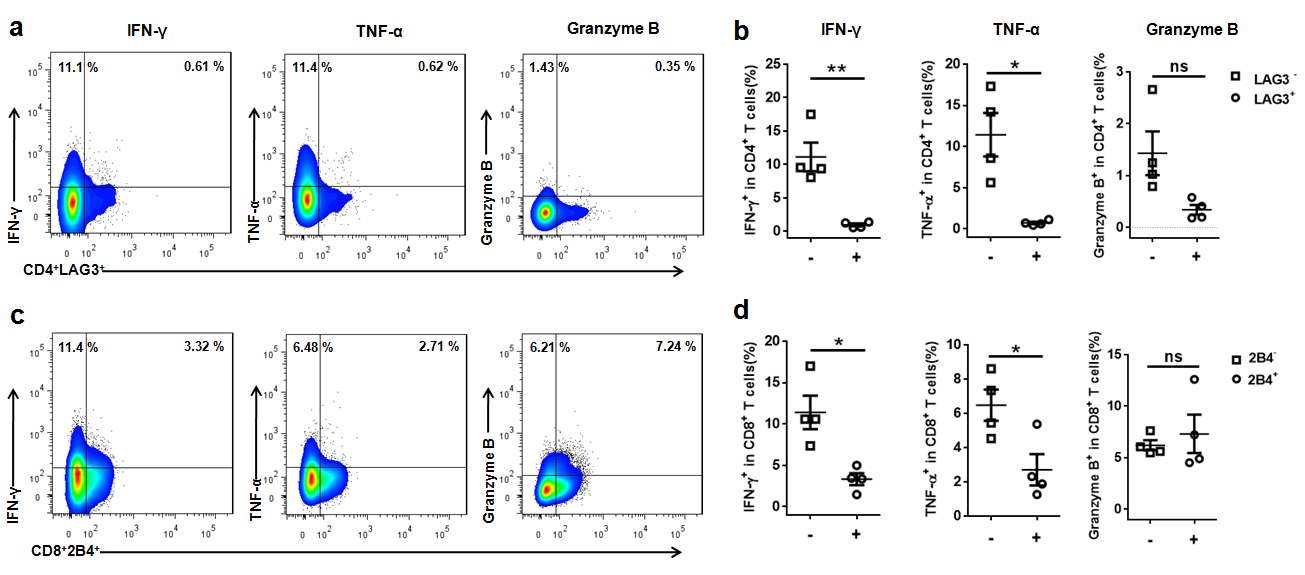
**

**Supplementary Figure 8. Overall changes of T cell subsets and related cytokine profiles in the liver from mice infected with different *E. multilocularis* PSC inocula during the course of infection.**


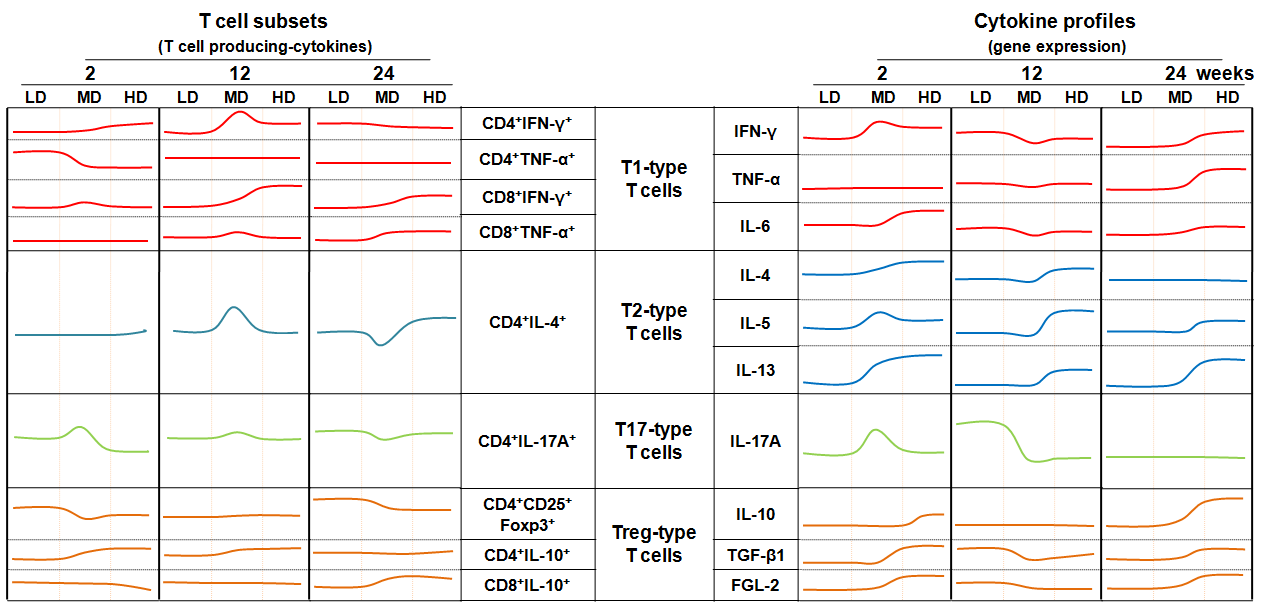

Supplement: Supplementary file 1 — Supplementary info [file 41598_2017_11703_MOESM1_ESM.doc]
